# Relationship between Molecular Structure and Heat-Induced Gel Properties of Duck Myofibrillar Proteins Affected by the Addition of Pea Protein Isolate

**DOI:** 10.3390/foods11071040

**Published:** 2022-04-03

**Authors:** Xueshen Zhu, Jiaxin Zhang, Shaohua Liu, Ying Gu, Xiaobo Yu, Feng Gao, Renlei Wang

**Affiliations:** 1 Key Lab of Biological Functional Molecules of Jiangsu Province, College of Life Science and Chemistry, Jiangsu Second Normal University, Nanjing 211200, China; xueshen_zhu@163.com (X.Z.); jiaxinzhang305@163.com (J.Z.); bclsh@jssnu.edu.cn (S.L.); joycemvp2012@163.com (Y.G.); 2 College of Animal Science and Technology, Nanjing Agricultural University, Nanjing 210095, China; 3 College of Food Science and Technology, Nanjing Agricultural University, Nanjing 210095, China; yuxiaobo@njau.edu.cn

**Keywords:** pea protein isolate, duck myofibrillar protein isolate, gel proprieties, heat-induced gel, molecular structure

## Abstract

This paper investigates the relationship between the molecular structure and thermally induced gel properties of duck myofibrillar protein isolate (DMPI) as influenced by the addition of pea protein isolate (PPI). The results showed that b* value of the gels increased; however, a* value decreased with the increase of PPI content (*p* < 0.05). The whiteness of the gels decreased significantly with the addition of pea protein compared with 0% vs. 0.5% addition. Nuclear magnetic resonance tests showed the area of immobilized water also increased with increasing PPI addition (0–2%), thus consistent with the increased water-holding capacity (*p* < 0.05). The penetration force of the gels increased with increasing PPI addition (*p* < 0.05), while the storage modulus and loss modulus of the gels were also found to increase, accompanied by the transformation of the α-helix structure into β-sheet, resulting in better dynamics of gel formation. These results indicated the gel-forming ability of DMPI, including water retention and textural properties, improves with increasing PPI addition. Principal component analysis verified these interrelationships. Thus, pea protein could improve the properties of duck myofibrillar protein gels to some extent and improve their microstructure, potentially facilitating the transition from a weak to a non-aggregated, rigid structure.

## 1. Introduction

Duck is a superior product even over lamb, beef, and pork because of its high nutritional value, including the presence of more polyunsaturated fatty acids and its relatively low price. Duck production is likely to play a significant role in the agricultural economy. Asian countries, especially China, account for 84.2% of the world’s total duck meat production [1]. Even though many traditional products, including Beijing roasted duck, Nanjing salted duck, pressed salted duck, and soy sauce duck neck, are widely preferred and consumed in China, they do not satisfy consumer demand for duck products [2]. In response to the new consumption trend, the food industry is constantly searching for cheaper and healthier protein components to replace that from animal sources. At the same time, some consumers have a negative perception of red meat because they are concerned that excessive red meat intake can lead to an increased incidence of metabolic diseases, such as cardiovascular disease [3]. Therefore, proteins of plant origin are gaining attention as alternatives to animal proteins. Pea protein is an emerging agricultural product with great potential as a functional ingredient for the food industry, which is widely utilized as an extender in milk replacement products, cereal, and bakery products and meat products, including sausage and meat patties [4]. It has a higher nutritional value than soy protein isolate and does not cause allergies [5]. Pea protein isolates can be used in various food applications related to heat-induced gels [4], but in general, the range of applications is narrow, and the value of pea protein resources has not been effectively exploited, especially when added to meat products [6]. On the other hand, it is well-known that pea protein has good solubility, foaming capacity, foam stability, and emulsification properties, and its total or partial addition can optimize the nutritional composition of the finished product, improve texture, and enhance its stability [7]. In addition, except for the low methionine content, pea protein has a balanced ratio of other amino acids [8,9] and can basically be called a full-valent protein with functions such as gastrointestinal regulation, lowering blood pressure, and improving immunity [10]. When proteins of plant origin are added to meat products, their nutritional composition and dietary fiber contribute to the improvement of the nutritional and quality characteristics of the product [11], while the production cost is reduced due to the increase in water-holding capacity [12]. However, to our knowledge, few studies have discussed the mixing behavior of duck myofibrillar protein (DMPI) with pea protein isolate (PPI) and its thermal gel properties. A deep understanding of the gelling mechanism of DMPI–PPI at different additions would improve the utilization of pea protein isolate for the development of healthy meat products with various properties.

The purpose of this study was to effectively utilize pea protein as an extender for gels formed from duck myofibrillar protein, which brings greater potential for expanding the use of healthier and more convenient foods and meeting consumer demand for a variety of food matrices. To this end, we substituted duck myofibrillar protein with pea protein isolates to varying degrees, aiming to investigate the relationship between molecular structure and thermally induced gel properties.

## 2. Materials and Methods

### 2.1. Sample Collection and Chemicals

Pea protein was obtained from Yantai Oriental Protein Technology Co., Ltd (Yantai, China). with 80% protein content; fresh duck breast muscle was taken from the local farmers’ market in Nanjing, Jiangsu Province. The fat and white fascia were removed from the breast meat as much as possible. The duck breast meat was then packed in self-sealing bags and sent to the laboratory, where it was stored in a refrigerator at −80 °C for freezing and storage. All chemical reagents were chemically pure.

### 2.2. Preparation of Heat-Induced Mixed-Protein Gels

The extraction of myofibrillar proteins from duck breast was appropriately modified according to the method of Park et al. [13] with minor modification. A bag of 30 g of duck breast was weighed, about 120 mL of extraction solution (four times the volume) was added to the meat grinder and stirred well, and the slurry was transferred to a 50 mL centrifuge tube and dispersed with a disperser at 10,000× rpm for 30 s. The slurry was then centrifuged at 4 °C for 10 min at 4000× *g*, repeatedly washed three times, filtered through gauze a second time, washed with 1% Triton X-100 for the above precipitate twice, centrifuged at 4000× *g* for 10 min, washed three times repeatedly with 4 times the volume of 0.1 mol/L NaCl, centrifuged at 4000× *g* for 10 min at 4 °C, and finally filtered through two layers of gauze to collect the precipitated myofibrillar proteins for backup. The precipitate (0.25 g) was placed in a 5 mL centrifuge tube, dissolved with 0.6 mol/L NaCl (containing 10 mmol/L K_2_HPO_4_, pH 7.0), and kept at 4 °C. The concentration of myofibrillar protein was then measured by Biuret method, and the final concentration was adjusted to 40 mg/mL. Different amounts of pea protein isolate were added (0, 0.5, 1.0, 1.5, and 2.0%, *w*/*v*) and dispersed. The protein mixture was then transferred to a small 10 mL beaker. Three replicates were set up for each group. After heating in a water bath (75 °C for 35 min), the gels were cooled with ice water for 30 min and chilled in a refrigerator at 4 °C for 12 h.

### 2.3. Color Measurement

Gel color was measured with Minolta CR-400 (illuminant D65) equipment (Minolta Camera, Osaka, Japan), calibrating with a standard plate before use (L* = 28.97, a* = 0.47, b* = −0.30), and the brightness (L*), redness values (a*), and yellowness values (b*) were recorded, and the average value was obtained by repeating six times. The whiteness values were calculated as follows [14]:

$$\mathrm{Whiteness}=100\sqrt{{\left(100-L^{*}\right)}^{2}+a^{*}+b^{*}{}^{2}}$$

### 2.4. Water-Holding Capacity

The water-holding capacity (WHC, %) was measured with concerning the method of Kocher and Foegeding [15] with minor modifications. The mixed-protein gels were weighed and centrifuged at 4 °C and 5000× *g* for 10 min. Inverted for 20 min, excess water was removed with filter paper, and the mass of the gel was weighed before centrifugation and in the centrifuge tube; *W*_1_: weight of the sample and centrifuge tube before centrifugation (*g*); *W*_2_: weight of the sample and centrifuge tube after centrifugation (*g*); *W*: weight of the centrifuge tube (*g*).

$$\mathrm{WHC}=\frac{W_{2}-W}{W_{1}-W}\times 100\%$$

### 2.5. Gel-Penetration Test

Gel-penetration force was measured with a texture analyzer (TA-XT plus Plaser, Stable Micro System, Surrey, UK). Measurement conditions: p/0.5R probe, pre-test rate of 1 mm/s, test rate of 0.5 mm/s, post-test rate of 10 mm/s, compressed mode selected, probe depth distance of 5 mm, and trigger force of 5 g, with 3 repetitions for each treated sample.

### 2.6. Low Field NMR Measurements

Three grams of samples were placed into a test bottle (15 mm diameter × 30 mm height), and the T_2_ relaxation time of the sample was determined using an NMR analyzer (MesoMR23-060H-1, Niumag electric Co., Shanghai, China). Experimental parameter setting: Carre Purcelle Meiboome Gill, proton resonance frequency of 22.6 mHz, the number of collected echoes was 3000. The NMR data were primarily analyzed using discrete exponential fitting and continuous distribution inverse. Each sample was analyzed six times.

### 2.7. Raman Spectroscopy Measurements

Raman spectra were determined using a Labram HR800 spectrometer (Horiba Jobi Yvon S.A.S., Longjumeau, France) according to the method of Zhuang et al. [16] with minor modifications. Raman spectra of the mixed-protein solution were collected in the 400–3600 cm^−1^ under the following conditions: three scans, exposure time 30 s, resolution 2 cm^−1^, sampling speed 120 cm^−1^. min^−1^, and data acquisition speed 1 cm^−1^. The spectra were standardized with the phenylalanine band at 1003 cm^−1^ by Labspec version 5 (Horiba Jobi Yvon S.A.S., Longjumeau, France). The content of protein secondary structure was calculated by Alix’s method according to the change of amide I band [17].

### 2.8. Dynamic Rheological Measurements

The rheological properties of DMPI-PPI mixed proteins were measured using a rheometer (MCR-301, Anton Paar, Graz, Austria) in oscillatory mode. Parameter setting: the selected fixture was 50 mm plate, the gap between the upper and lower plates was 1 mm, the frequency was 0.1 Hz, the strain was 2%, the temperature was raised from 30–80 °C at the speed of 2 °C/min, and the cooling rate was 5 °C/min. Before the test, a drop of paraffin oil was added to the edge of the plate to separate the sample from the outside air and prevent the sample from evaporating due to heat. The energy storage modulus (G’) and loss modulus (G″) were recorded, and tan δ was then calculated according to the ratio of G′ and G”.

### 2.9. Microstructural Analysis

Scanning electron microscopy was used to observe the morphology of the mixed-protein gel referring to the method of Jiang et al. [18]. The heat-induced protein gel was cut into small pieces (5 × 5 × 1 mm) and fixed with 2.5% glutaraldehyde for 24 h. Each sample was freeze-dried and sputter-coated with 10 nm of gold. Gels were then analyzed with a Hitachi S-3000N scanning electron microscope (Tokyo, Japan) at an accelerating voltage of 20 kV. The microscopy images were then analyzed with the public domain software ImageJ v1.52a (Rawak Software Inc., Stuttgart, Germany) to measure the average cavity size.

### 2.10. Statistical Analysis

One-way analysis of variance (ANOVA) and Duncan’s multiple range test for statistical analysis was conducted using SPSS^TM^ software(version 20, SPSS Inc., Chicago, IL, USA). Principal component analysis (PCA) was performed to estimate the relationship between the DMPI–PPI protein structure and gel textural quality characteristic using SPSS^TM^ software (version 20, SPSS Inc., Chicago, IL, USA).

## 3. Results and Discussion

### 3.1. Gel Color

**Figure 1:**
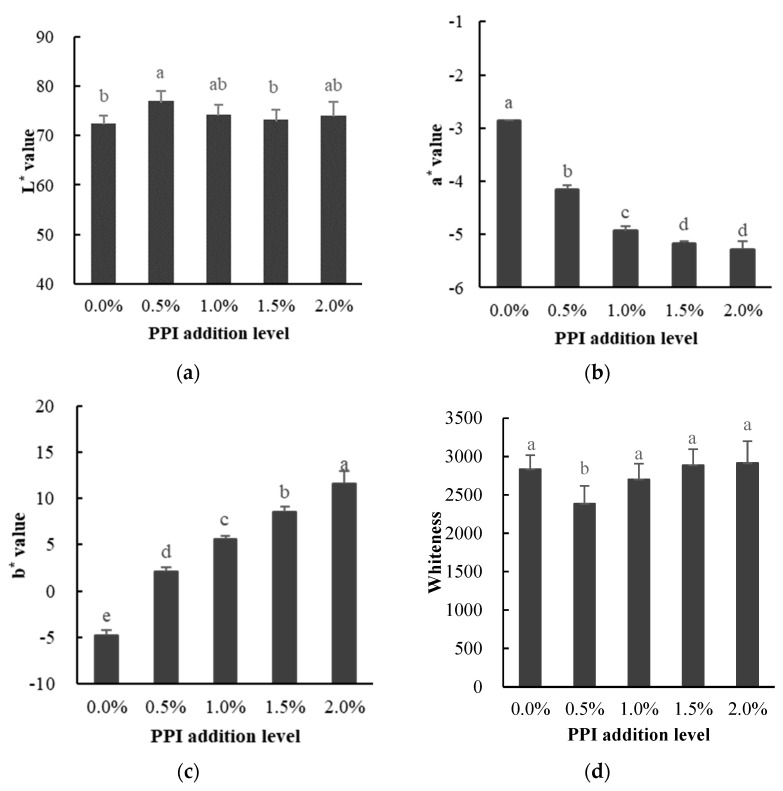
Effect of addition of pea protein on the L* value (**a**), a* value (**b**), b* value (**c**), and whiteness (**d**) of DMPI–PPI gel. Note: 0%, 0.5%, 1.0%, 1.5%, and 2.0% indicate level of addition of PPI to DMPI. Different letters (a–e) indicate significant difference (*p* < 0.05).

Color is one of the important indicators to evaluate the sensory quality of meat products, and it will affect the purchasing intention of consumers to some extent. As seen in Figure 1a, the 0.5% PPI-addition group exhibited a significant higher brightness (L* value) compared to the control group (0% PPI addition) although no significant differences were found between the other groups; with the addition of pea protein, there was a definite increase in the L* value of the gel. The L* value shows the light scattered by the structural elements that make up the gel network (protein aggregates, molecular fragments, etc.). The matrix became denser when myofibrillar protein was partially added to the pea protein isolate. The redness (a* value) of the gels decreased with the addition of pea protein (*p* < 0.05), with the largest a* value for the gels with 0% PPI addition shown in Figure 1b. However, the difference between PPI additions of 1.5% and 2% was not significant. As seen in Figure 1c, the yellowness (b* value) of the gels increased significantly (*p* < 0.05) with increasing PPI addition, and it reached a maximum value of 11.58 (2% PPI addition). Since the color of pea protein isolate varies from creamy to beige depending on the processing method [7], the color of the gel is easily affected with increasing PPI addition. As seen in Figure 1d, the whiteness of the gel decreased significantly compared to the 0.5% addition. Although an increasing trend can be found with increasing addition from 0.5% to 2%, no significant differences were found between the 1%, 1.5%, and 2% PPI addition groups. This result is consistent with the findings of Borderías et al. [19], where the increase in b* values was associated with the presence of phenolic compounds, including anthocyanins and flavonols, which provide the characteristic pale yellow color of PPI. Then, the color properties of these compounds suggest that they absorb part of the light wave, which also leads to a decrease in the whiteness of the mixed gels.

### 3.2. Gel WHC

**Figure 2:**
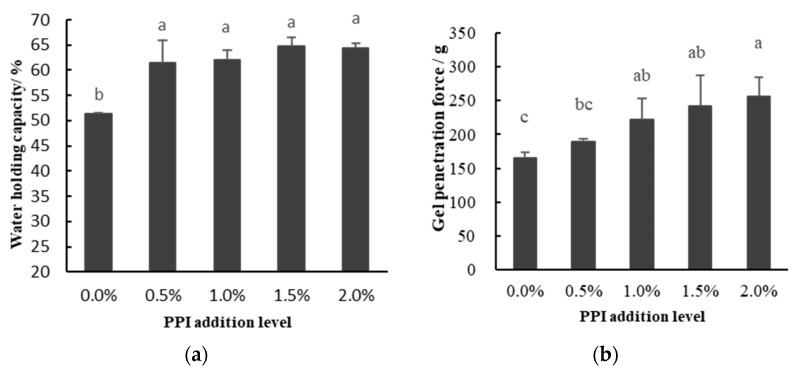
Effect of pea protein addition level on the water holding capacity (**a**) and gel penetration force (**b**) of DMPI–PPI mixed gel. Note: 0%, 0.5%, 1.0%, 1.5%, and 2.0% indicate level of addition of PPI to DMPI. Data of WHC are expressed as the mean ± SD. Different letters indicate a significant difference (*p* < 0.05), and each treatment was performed in triplicate (*n* = 3).

It is widely accepted that a gel can be considered as a network in which the “grid” of the network is filled with water molecules. Water retention reflects the water changes in the gel network caused by capillary effects in the mixed-protein matrix [20]. Figure 2a shows the effect of PPI addition on the water retention of the mixed-protein gels. There were significant differences in water retention between the control group (PPI addition of 0%) and the experimental groups (addition of 0.5–2%), but no significant difference was found within the experimental group (addition of 0.5–2%). Li et al. [21] found that as the amount of chickpea protein isolates added increased from 0 to 15 g/kg, the WHC of pork myofibrillar protein gels increased from 75.85–86.30% (*p* < 0.05) significantly. These results are consistent with other studies that have shown a significant increase in WHC of meat products through the addition of non-meat proteins [22] and polysaccharides [23]. This trend of increased WHC suggests that a more favorable physical capture of water occurs in mixed molecular matrices and that higher additions promote the capture of water molecules [24]. From our results, it appears that water may be kept in the lattice of the myosin network or bound to the functional groups of PPI. As is well-known, that the WHC of gels depends essentially on the structural stability of the gel [25]. The enhanced intermolecular interactions upon heating and the more compact and homogeneous gel structure induced by PPI, which will be mentioned later, may also have a conductive effect on the entrapment of water in the gel network. The increase of WHC in DMPI–PPI gel is partly due to these factors.

### 3.3. Gel-Penetration Test

Gel-penetration force (GPF) reflects the gel strength of the protein, which is an important indicator related to the gel texture and depends on the variation of the gel microstructure [26]. A high gel strength reflects the formation of a gel structure that is compact, stronger, and stable. The effect of PPI supplementation on the gel penetration of DMPI is shown in Figure 2b. Gel-penetration force of DMPI gradually increased with increasing PPI addition (0–2%), but the rising trend decreased, as no significant differences were found between the groups with 1%, 1.5%, and 2% addition. However, results also showed significant differences between the 0.5% and 2% mixed-gel groups were found. These results suggest that PPI can improve the gel strength of DMPI to some extent. It is speculated that addition of PPI helps DMPI to form a filled-gel system where pea proteins are filled into the gel network formed by myosin, making the gel network denser and eventually showing an increase in protein gel-penetration force. Wang and Damodaran [27] also correlated the gel strength with the degree of protein denaturation and unfolding under given conditions, with the greater the degree of protein denaturation and unfolding the stiffer the gel. Previous studies have also shown that the polysaccharide fraction can significantly increase the gel stiffness of myofibrillar protein gels [28], in addition, the elasticity of myofibrillar protein gels was also significantly improved [29]. As pea protein enhances emulsification and gelation, the number of molecules per unit volume and their intermolecular collision probability increase with the increase of PPI addition, forming a stable gel network structure. Gel elasticity generally increased first with increasing soy protein isolate content, which is similar to the results of Jiang et al. [30]. Niu et al. [31] suggested that the increase in gel strength may be attributed to the regular arrangement of the protein network. Thus, when non-meat protein is applied to ground meat as a potential texture modifier, it can be effective at relatively low concentrations. Sun et al. [12] reported that the addition of peanut protein isolate to chicken salt-soluble protein improved the gel strength of thermally induced mixed-protein gels, as peanut protein isolate may be used as a meat binder. However, it is worth mentioning that when excessive amounts of non-meat proteins are added, they instead hinder the three-dimensional network structure of myofibrillar protein gels and reduce their gel strength [26]. Mccord et al. [11] also reported penetration force of salt-soluble muscle proteins gel decreased when excessive amounts of native soy protein isolate were added, and the degree of decrease was proportional to the amount of added.

### 3.4. NMR Analysis

**Figure 3:**
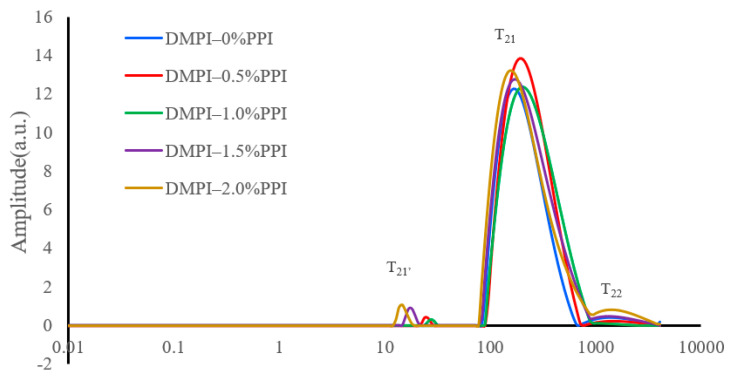
Effect of pea protein addition level (0%, 0.5%, 1%, 1.5%, and 2%) on the distribution of the T_2_ of DMPI–PPI mixed gel. T_2_, spin-spin relaxation times (ms) for different types of water. Each treatment was performed in triplicate (*n* = 3). T21’: a small population (or populations) at approximately 10 ms or less; T21: a dominant population between 80–000 ms; T22: a minor peak after 1000 ms represents free water.

To further investigate whether the addition of pea protein stabilizes water in the composite duck myofibrillar protein gels, T_2_ relaxation measurements were performed. With this approach, three major characteristic peaks were observed in the heat-treated mixed-protein gels (Figure 3); a small population (or populations) at approximately 10 ms or less (often referred to as T_21_’, partially immobilized water [32], and a dominant population between 80–000 ms (denoted T_21_) [33] and a minor peak after 1000 ms represents free water T_2__2_. These results suggest that during heating, myofibrillar proteins inter cross-linked, forming a network structure that locks a large amount of free water into it, making it immobilized. The area of total immobilized water (including T_21_’ and T_21_) was significantly increased in the experimental group (PPI addition of 0.5–2%) compared to the control group (PPI addition of 0%); however, no significant difference was found within the experimental group. Previous studies also reported similar results that the relaxation time of the composite gels and the relaxation peak area of free water was reduced after the addition of 1.5% chickpea protein isolate [21]. Compared with the control, the addition of 1.5% water insoluble dietary fibers significantly increased the immobilized water of gel and was accompanied by a remarkable decrease in free water [25]. These results revealed that the addition of pea protein stabilizes the water distribution in the composite gels and that the interaction between myofibrillar protein and pea protein powder in the network structure is enhanced [34]. The area of free water T_22_ decreased. This suggests that the addition of pea protein helps to convert free water into immobilized water and reduce the amount of free water [35]. The water retention of myofibrillar protein gels could be enhanced by the addition of pea protein since the free water content is inversely proportional to the water retention [36]. These results can be consistent with the above-mentioned results of WHC.

### 3.5. Rheological Properties Analysis

**Figure 4:**
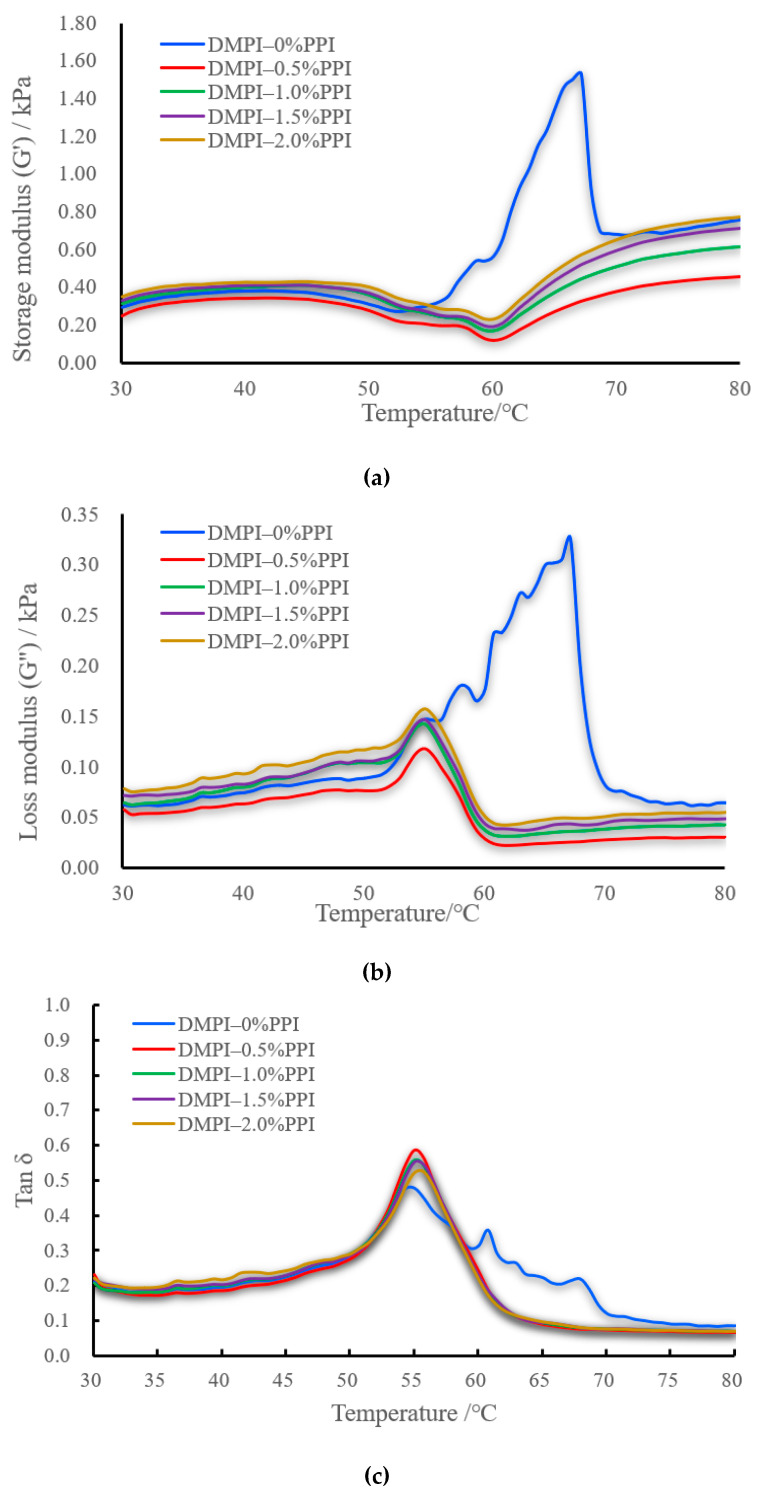
Effect of pea protein addition level (0%, 0.5%, 1%, 1.5%, and 2%) on the storage modulus (**a**), loss modulus (**b**), and phase angle/tanδ (**c**) of DMPI-PPI mixed gel. Each treatment was performed in triplicate (*n* = 3).

The storage modulus (G’) of DMPI–PPI mixed proteins can reflect not only the unfolding and agglutination process of protein molecules at different temperatures but also the elasticity and gelation of protein gels [30]. As shown in Figure 4, the storage modulus trend of DMPI with pea protein addition was consistent throughout the heating process. As the pea protein addition increased from 0.5–2%, the final G’ value also increased, which is consistent with the gel-penetration force results, indicating that the addition of pea protein contributed to the formation of duck myofibrillar protein gels and improved the qualitative characteristics of the aforementioned gels. In the range of 30–45 °C, the storage modulus increased slowly, which may indicate that the gel network was formed at the beginning. At this time, myosin began to cross-link through the interactions between the head dimers and slowly formed the elastin network structure. At 45 °C, the storage modulus began to decrease, which was relatively flat from 52–58 °C and started to decrease after 58 °C. At 60 °C, the storage modulus reached its lowest value. The sharp decrease may be related to the expansion of the myosin tail. Thereafter, G’ rises continuously in the range of 62 °C and 80 °C, reaching a maximum of 80 °C [37]. The storage modulus of the gels without pea protein was the same as that of the gels with pea protein until 52 °C, but after that, it did not decrease but rather increased. A small peak was reached at 58 °C and then continued to rise. This may be due to the instability of the gel structure formed when no pea protein was added. In addition, pea vicilin reduced the self-aggregation of myosin heavy chains during heating and delayed the temperature of myosin head denaturation after reaching the peak [38], partly because the addition of pea protein could delay the temperature of myosin tail denaturation during gel formation [39]. However, it is also evident that the final G’ and final G” values of the different experimental groups (0.5–1.5% PPI addition) were lower than those of the control group (0% PPI addition), suggesting that PPI can cross-link with MPI during programmed heating (30–80 °C at a rate of 2 °C/min) but with limited effect [38]. Compared to the above results for gel strength and water retention, thermostatic heating (heating at 75 °C for 35 min) was more favorable than the programmed heating described above for the formation of mixed-protein gels, which may contribute to the denaturation of pea vimentin and bean protein [6]. Stiffer gels can even be formed during constant temperature heating; however, the lack of interference with protein–protein interactions responsible for the formation of less elastic structures has a detrimental effect on muscle protein gelation. Higher tanδ values, which indicate a more viscous or less elastic sample [40], gradually increase as the heating temperature increases from 20–55 °C, followed by a sharp decrease in all these treatments (Figure 4c). These changes indicate that viscosity of DMPI–PPI mixed proteins is higher than elasticity at the beginning of heating.

### 3.6. Changes in the Protein Secondary Structures

**Figure 5:**
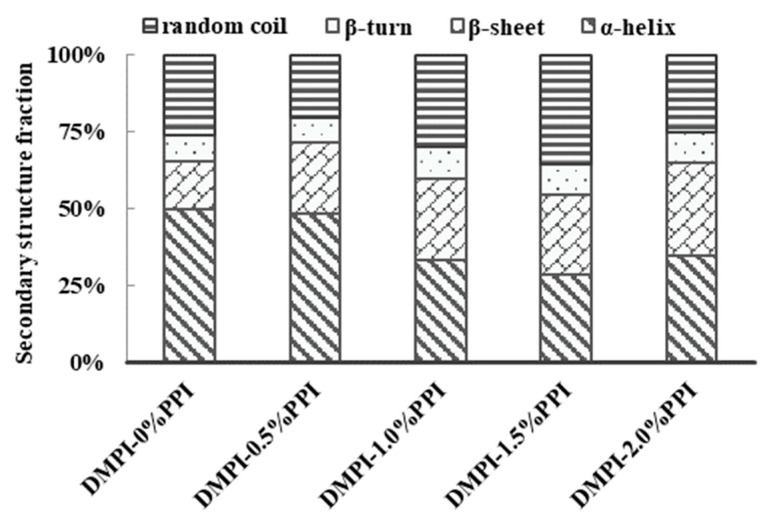
Effect of pea protein addition level (0%, 0.5%, 1%, 1.5%, and 2%) on the change of protein second structure of DMPI–PPI mixed gel. Each treatment was performed in triplicate (*n* = 3).

The Raman bands of muscle fibers in the range of 1600–1700 cm^−1^, especially the band at 1654 cm^−1^, can reflect the vibrational modes of amide I, which mainly involves C = 0 stretching and N–H bending of a small fraction of peptide groups [17]. The exact position of these bands and the corresponding secondary structures of DMPI-PPI gels were quantified in this study to obtain Figure 5. Thus, in general, the α-helix, β-sheet, and random coil structures correspond to the 1658–1650, 1680–1665, and 1665–1660 cm^−1^ ranges of the amide I band, respectively [41]. It clearly showed that the addition of pea protein led to a change in the amide I band in the Raman spectrum of the mixed protein, with a decrease in the α-helix content; however, content of β-sheet increased with the addition of PPI. Similar results also showed that thermal processes induced a decrease in α-helix content and an increase in β-sheet content during myofibrillar protein denaturation [42]. Pea proteins are reported to mainly consist of vicilin, 7 s and legumin, 11 s, which show a high endothermic conversion [38] and generally do not affect MP denaturation. Zhuang et al. (2021) also reported consistent results in the effect of konjac glucomannan on myofibrillar protein gels [43]. Thus, the addition of pea protein during thermal processing facilitates the unfolding of the total secondary structure in the mixture, with more α-helix unfolding, more exposure of hydrophobic groups, and the formation of a rigid gel network. The results fit well with the previous results of gel-penetration tests.

### 3.7. Principal Component Analysis

**Figure 6:**
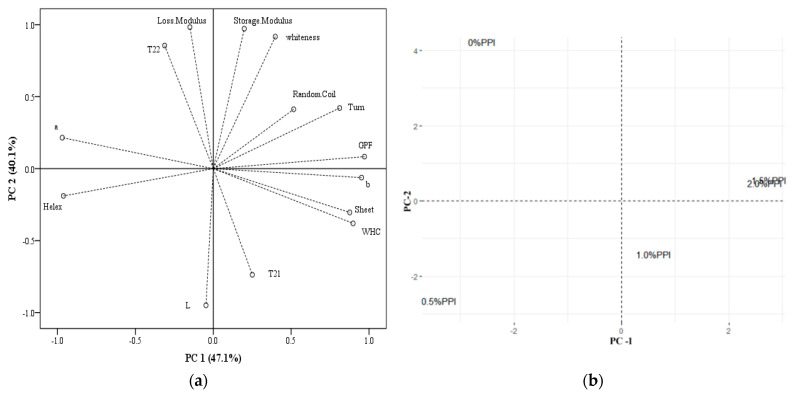
Loading plots (**a**) and score plots of groups (**b**) of PCA analysis at various level of addition of PPI (0%, 0.5%, 1%, 1.5%, and 2%).

The eigenvalue plots show the explanation of the overall variation by the two principal components, accounting for 47.1% and 40.1% of the variation in the data, respectively (Figure 6a). Thus, the model explains 87.1% of the total variance in the data, and these data show a strong correlation between the original data. As shown in Figure 6a, the first principal component (47.1% variation) was mainly positively correlated with WHC, GPF, α-helix, β-sheet, and β-turn and negatively correlated with a-values and α-helix, which were mainly related to the internal chemical forces and color of the gels. These results suggest that water mobility, GPF, and a-helix are the three key factors affecting the performance of DMPI–PPI gels. This may be due to the addition of pea protein isolate enhancing the water-binding ability and better gel strength of rigid gels. PC2 (40.1% variation) is characterized by four variants (final G’, final G”, T_21_, and T_22_). The score plot shows the group distribution of PC scores in multivariate space. As shown in Figure 6b, the DMPI–PPI gels are arranged from left to right along PC1 and are distinguished into four groups. It can be inferred that the water fluidity, color, and texture of the DMPI 1.5% PPI group were similar to those of the DMPI 2% PPI group. When excessive amounts of non-meat proteins are added, no additional improvement in gel properties may be found, and it may even hinder the network structure of the mixed-protein gels [26]. This suggests that the distribution of water is closely related to the viscoelastic changes during the dynamic protein-gelation process [25]. During gelation, free water is removed from the gel matrix, while the viscoelastic gel is transformed into an elastic gel, which helps to improve the gel properties.

### 3.8. Microstructure of the Gels

**Figure 7:**
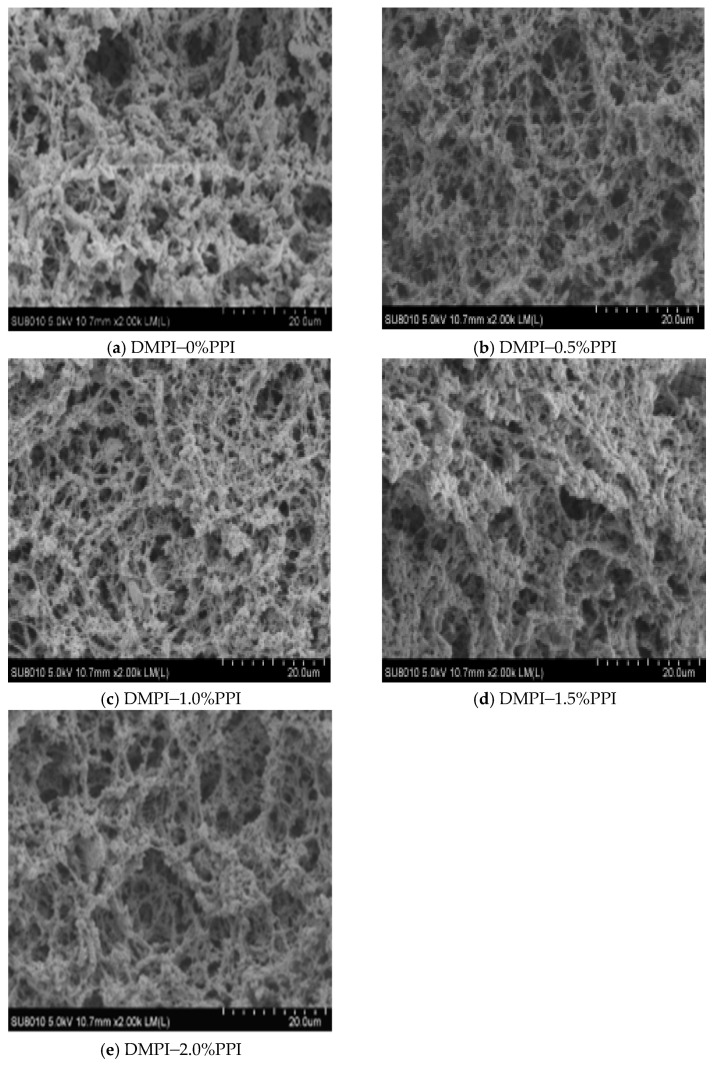
Effect of pea protein addition level (0%, 0.5%, 1%, 1.5%, and 2%) on the microstructure (SEM, 2000×) of DMPI–PPI mixed gel. Each treatment was performed in triplicate. (**a**) DMPI–0%PPI; (**b**) DMPI–0.5%PPI; (**c**) DMPI–1.0%PPI; (**d**) DMPI–1.5%PPI; (**e**) DMPI–2.0%PPI.

Protein isolation is widely used as a binder for meat products to improve yield and texture and as surfactants to improve emulsion stability during heating. As can be observed in Figure 7, although the individual MP gels showed a 3D network structure, the structure was not homogeneous, with significant large diameter pores and voids on the surface. However, the network structure of the experimental group containing pea protein (0.5–2%) is relatively compact, and the structure becomes more compact. Cavity size was measured using software ImageJ v1.52a (Rawak Software Inc., Stuttgart, Germany), and there are significant differences in cavity size among the 0%, 0.5%, and 1% groups, as the average cavity size obviously decreases (13.7, 11.59, 6.9 μm). However, no significant differences were found among 1% group, 1.5% group, and 2% group although a trend of decease (6.9, 6.0, and 5.5 μm) also existed. This may be due to the fact that pea protein molecules interspersed in the network structure, relying on the expansion of volume after water absorption, exerted repulsive and supporting effects on the protein network, filled the pores between proteins, promoted direct limited adhesion of proteins, made the gel network more compact and orderly, and improved the structure of the protein gel network [16]. In semi-compatible systems, non-gelling proteins can bind to networks of other proteins, reducing the flexibility of the network and producing much more rigid gels [44]. Several studies have found that non-meat proteins can enhance the ability of MPI to thermally induce gel formation and make the gel structure denser [28]. In general, the textural properties of the composition depend on the dispersed phase of the gel matrix, including the degree of dispersion and phase volume [45,46]. Isolated pea proteins can be used as a matrix in a second continuous phase or directly with muscle proteins in semi-compatible gels [22]. It was hypothesized that pea protein could promote the formation of bovine salt-soluble protein gel structures mainly through physical filling and that the addition of pea protein significantly reduced the particle size compared to the control (data not shown in our results), while the addition of pea protein would inevitably reduce the average particle size of the mixed proteins. The limited connection between filling, encapsulation, and mechanical support, which may be through drainage, is mediated by a chemical combination of hydrogen bonding, hydrophobic interactions, etc.

## 4. Conclusions

The gel-forming ability of DMPI, including water retention and textural properties, improves with increasing PPI addition. The addition of pea protein promotes the conversion of free water to immobilized water. α-Helix content decreases while β-sheet content increases upon heating, providing the driving force for gel formation and producing a more compact and homogeneous gel structure induced by PPI, which may also have a conductive effect on the entrapment of water in the gel network. While processing duck gel products, less than 2% of PPI is strongly recommended to utilize in order to increase gel strength and water retention; however, more attention should be paid to the effect on the color of products. Further studies are necessary to determine the effect of protein–protein interactions on gel properties to investigate the mechanism and provide additional opportunities to expand the utilization of pea protein in duck products.

## Data Availability

The datasets generated for this study are available on request to the corresponding author.
